# Tooth size discrepancy in orthodontic patients with skeletal anterior open bite

**DOI:** 10.1002/cre2.647

**Published:** 2022-08-17

**Authors:** Emad F. Al Maaitah, Nada Al‐Madani, Elham S. Abu Alhaija

**Affiliations:** ^1^ Department of Orthodontics, Faculty of Dentistry Jordan University of Science and Technology Irbid Jordan; ^2^ College of Dental Medicine, QU Health Qatar University Doha Qatar

**Keywords:** Anterior open bite, Bolton discrepancy, Skeletal, tooth size

## Abstract

**Objective:**

To find out if there is any relationship between tooth size discrepancy (TSD) and skeletal anterior open bite (AOB) and to assess the correlation between the amount of AOB and TSD.

**Method:**

A total of 100 Class I subjects were included in this study (average age 15.21 ± 2.84 years). Fifty patients had skeletal AOB (>3 mm) and 50 subjects acted as controls and had a normal overbite. Teeth mesio‐distal widths were measured using a digital caliper. Anterior, overall, and posterior TSD ratios were calculated. An independent *t‐*test was employed to assess differences between groups and between genders. Pearson correlation coefficient was used to assess the correlation between the amount of AOB and TSD.

**Results:**

Significant differences in anterior (*p* = .038) and posterior (*p* = .015) TSD ratios were detected. In the skeletal AOB group, no significant gender differences were detected (*p* > .05), whereas in the normal bite and total sample group, males had smaller posterior teeth compared to females (*p* < .05). All the differences were smaller than 1 SD of Bolton's ratios. No significant correlation was found between the amount of AOB and TSD ratios (*p* > .05).

**Conclusions:**

Skeletal AOB had larger anterior and smaller posterior mandibular teeth, but the differences were less than 1 SD of Bolton's ratios. Males have smaller mandibular posterior teeth than females. The amount of AOB is not correlated with the TSD ratios.

## 1 INTRODUCTION

Bolton has developed two ratios to estimate the tooth size discrepancy (TSD) based on a study of 55 cases of excellent occlusion. The ratio of summed mesio‐distal widths of the mandibular to maxillary teeth (from the first molar to the first molar for the overall ratio, or from canine‐to‐canine teeth for the anterior ratio) could be compared with standard values in order to quantify a patient's TSD (Bolton, 1958).

Bolton later considered that TSD is significant when a ratio greater than 1 standard deviation (SD) from the reported mean values exists (Bolton, 1962). However, other authors suggested that a significant TSD can be considered only when more than 2 SDs from Bolton's mean values exist (Crosby & Alexander, 1989; Freeman et al., 1996).

TSD was defined by Proffit as the disproportion among the size of individual teeth (Proffit, 2007). The interrelation between mesio‐distal teeth width and arch alignment has been of clinical interest. Dental crowding is associated with large teeth and it is considered the most common type of malocclusion in addition to other occlusal problems (Radnzic, 1988). In addition to the Andrews Six Keys of normal occlusion, some authors suggested TSD as the seventh important key that could affect normal occlusion as it causes spacing, crowding, and incorrect intercuspation (McLaughlin et al., 2001; Rakosi et al., 1993).

To ensure getting the best occlusal relationship; overbite, intercuspation, and overjet, a strict relationship between teeth size and the size of maxillary and mandibular arches should exist and between that of maxillary and mandibular teeth (Basaran et al., 2006).

The prevalence of TSD in the general population has been mentioned by Proffit as being 5% (Proffit, 2007). His definition considered cases that fall outside 2 SDs from Bolton's mean ratios as TSD. Othman and Harradine, in their literature review, reported that 20%−30% of people have a significant anterior TSD and 5%−14% have overall TSD (Othman & Harradine, 2006).

Correct diagnosis of TSD in the treatment planning stage will help the orthodontist to achieve perfect results during orthodontic treatment finishing. Bolton TSD ratios are the recommended golden standard diagnostic method for the identification of a TSD.

Anterior open bite (AOB) is defined as the lack of overlap in the vertical direction between the maxillary and mandibular anterior teeth with the posterior teeth in occlusion (Subtelny & Sakuda, 1964). The prevalence of AOB ranges from 1.5% to 11% and differs between ethnic groups, age, and dentition type (Ng et al., 2008). AOB tends to have a multifactorial etiology. Numerous theories have been proposed, including heredity, unfavorable growth patterns, digit sucking habits, mouth breathing, and tongue function (Alexander, 1999).

The relationship between AOB and TSD has not been studied extensively before, although both have been linked to different types of malocclusion and different occlusal traits.

To the best of our knowledge, only one study was found assessing the relationship between TSD and dental AOB (Salwa, 2016). Taibah, in her study of the Saudi sample, looked at TSD and dental AOB and found no differences between AOB and control groups in any of the TSD ratios (Salwa, 2016). However, neither dental nor skeletal relationships were mentioned for the included subjects.

Grauer and his colleagues mentioned some clinical findings that could be related to TSD, including incisors spacing or crowding, increased or decreased overjet, excessive or deficient overbite, excessive prominence of the upper incisors and canines marginal ridges, and abnormally angulated or inclined incisors and canines (Grauer et al., 2012). It is well known that these clinical findings are not specifically correlated to TSD, but could be found in other types of malocclusions such as AOB.

Interarch TSD can preclude obtaining a balanced occlusion with good inter digitation, as well as appropriate overjet and overbite (Araujo & Souki, 2003).

This study will help to shed a light on the relationship between skeletal AOB and TSD and if there is any correlation between the amount of AOB and TSD.

Accordingly, the aim of the present study was to find out if there is any relationship between TSD and skeletal AOB in Class I malocclusion patients and to look for any correlation between the amount of AOB and TSD.

## 2 MATERIALS AND METHODS

### 2.1 Study design

This was a retrospective cross‐sectional study carried out on the available pre‐orthodontic treatment study models and lateral cephalograms of two groups of patients; the first group was patients with skeletal AOB and the second with a normal overbite who acted as a control group. Pre‐orthodontic treatment study models and lateral cephalograms were collected from the archive of the orthodontic clinic at the dental teaching center and dental teaching clinics of Jordan University of Science and Technology. Ethical approval for the conduction of this study and access to patients' files was obtained from the Institutional Review Board (IRB) at Jordan University of Science and Technology.

### 2.2 Sample size

The sample size was calculated using the G*power 3.1.9 program. A total sample size estimate of 100 patients (50 subjects per group) was determined assuming an effect size difference of (0.5) between groups at a conventional *α* level (.05) and power (1– *β*) of .80.

### 2.3 Subjects and selection criteria

A total number of 100 Caucasian patients (average age 15.21 ± 2.84 years) were included in this study and divided into two groups according to the presence or absence of skeletal AOB. Included patients were selected consecutively from the available pretreatment records of orthodontic patients who met the inclusion criteria.

Group 1: consisted of 50 patients (24 males, 26 females) with Class I malocclusion (ANB 2−4°) and skeletal AOB (≥3 mm, maxillary mandibular planes angle [MMPA] > 32°, posterior facial height to anterior facial height ratio [PFH/AFH] is less than 59%).

Group 2: acted as a control and consisted of 50 patients (18 males, 32 females) with Class I malocclusion (ANB 2−4°) and normal overbite (1 < OB ≤ 3 mm, average MMPA 27 ± 5°, PFH/AFH> 59%).

Both groups were matched in age and type of occlusion.

All patients were selected according to the following inclusion criteria:

1. Age between 14 and 30 years old.
2. All permanent teeth erupted (except third molars).
3. No missing, extracted, or supernumerary teeth.
4. No abnormally sized or shaped teeth.
5. Minimal or no tooth wear.
6. Medically fit patients.
7. In the first group, AOB of at least 3 mm (skeletal; MMPA > 32°, PFH/AFH < 59%).
8. In the control group, normal over bite (1 < OB ≤ 3 mm, average MMPA, PFH/AFH > 59%).
9. Both groups have Class 1 skeletal (ANB 2−4°) and dental relationships.
10. No previous orthodontic treatment.
11. Bite registration when patients' dental impressions were taken is essential.

Accordingly, the following were the exclusion criteria:

1. Broken study models.
2. Mesiodistal composite restorations, gross restorations, crowns, onlays, or Class II amalgams.
3. Congenitally missing, impacted, and grossly carious teeth.

Lateral cephalograms were used to confirm the skeletal nature of AOB (MMPA > 32° and PFH/AFH is less than 59%) in Group 1, and the skeletal relationship of Class 1 in the two groups (ANB angle between 2° and 4°).

### 2.4 Measurements of the study models

The measurements were carried out directly on the study models using a digital caliper (Tresna instruments, series: SC02, ID: 111‐200‐10, Guillin, Guangxi Province, P.R. China) with a range of 0−100 mm and accuracy of 0.03 mm, resolution of 0.01 with fine tips to facilitate the access into the interproximal space. The mesio‐distal width of each tooth was measured at its greatest interproximal distance; from its mesial contact point to its distal contact point, from the first molar to the first molar in both arches, perpendicularly holding the caliper to the long axis of each tooth. All measurements were done by the same examiner (N. M.) for the whole sample. To make sure that the examiner was blinded for the group of study models (whether AOB or normal bite), each jaw was measured separately on different occasions to avoid any bias in the measurements.

All the measurements of each tooth from the first molar to the first molar were then transferred to the data sheets. The overall sum of maxillary and mandibular teeth mesio‐distal width (6 to 6) and the sum of the anterior maxillary and mandibular teeth mesio‐distal width (3 to 3) were calculated using the Microsoft Excel program.

Overall, anterior and posterior Bolton's TSD ratios were then calculated according to the following equations published by Bolton (Bolton, 1962).

$$\frac{\mathrm{Sum}\;\mathrm{of}\;12\;\mathrm{lower}\;\mathrm{teeth}\;(6-6)}{\mathrm{Sum}\;\mathrm{of}\;12\;\mathrm{upper}\;\mathrm{teeth}\;(6-6)}\;\;\;100\%\;=\;\text{overall ratio},$$

$$\frac{\mathrm{Sum}\;\mathrm{of}\;6\;\mathrm{lower}\;\mathrm{teeth}\;(3-3)}{\mathrm{Sum}\;\mathrm{of}\;6\;\mathrm{upper}\;\mathrm{teeth}\;(3-3)}\;\;\;100\%\;=\;\text{anterior ratio},$$

Overallratio – anteriorratio = posteriorratio.

### 2.5 Error of the method

Method error was assessed by randomly selecting the study models and lateral cephalograms of 10 subjects (10% of the sample) and re‐measuring them after an interval of 2 weeks by the same examiner (N. M.).

Dahlberg's formula (ME *=* √∑*d* ^*2*^ */2N*) (Dahlberg, 1940) for double measurements was used to calculate the method error.

Dahlberg formula was applied to the overall Bolton's discrepancy ratio, which represents the 24 mesio‐distal width measurements of the upper and lower teeth from the first molar to the first molar. Dahlberg error was found to be less than 0.6 for all the measurements, indicating no significant random error.

Dahlberg formula was also applied to the amount of AOB and the lateral cephalogram measurements: ANB, MMPA, and PFH/AFH ratio; Dahlberg error was found to be less than 0.8 for all the measurements, indicating no significant random error.

### 2.6 Statistical analysis

Statistical analysis was performed using the Statistical Package for Social Science (SPSS) computer software (SPSS 22.0, SPSS Inc., NY, USA). Descriptive and analytical statistics were employed. Descriptive data are described as mean ± standard deviation (SD). Independent *t* test was employed to assess differences between the two groups. To determine whether there were gender differences in the tooth size ratios and discrepancies among the AOB group or the control group, again independent *t* test was applied. Pearson's correlation coefficient was used to assess the correlation between the amount of AOB and TSD. The significance level was set to *p* < .05.

## 3 RESULTS

Table 1 shows the details of the study sample. The two groups were matched in terms of age and skeletal relationship (using ANB angle).

**Table 1:** Sample distribution

	AOB	Control	Total
Skeletal measurements			
SNA (°)	83	81	82
SNB (°)	79	78	79
ANB (°)	4	3	4
MMPA (°)	35	28	30
PFH/AFH (%)	58	63	60
Age			
Mean(SD)	16.04 (2.94)	14.92 (2.32)	15.21 (2.84)
Female	26 (52%)	32 (64%)	58
Male	24 (48%)	18 36%)	42
Total	50 (100%)	50 (100%)	100

Abbreviations: AFH, anterior facial height; ANB, A point‐Nasion‐B point; AOB, anterior open bite; MMPA, maxillary mandibular planes angle; PFH, posterior facial height; SNA, Sella‐Nasion‐A point; SNB, Sella‐Nasion‐B point.

Significant differences in anterior (*p* = .038) and posterior (*p* = .015) TSD ratios were detected between the two groups. In contrast, the overall TSD ratio showed no significant difference between the two groups (*p* = .368), Table 2.

**Table 2:** Means and standard deviation of TSD ratios in skeletal AOB and control groups

	AOB			Control			*p* Value
	Mean (SD)	Range		Mean (SD)	Range		
		Min	Max		Min	Max	
Overbite (mm)	−4.5 (−1.40)	−7.32	−3.18	2.8 (1.64)	1.52	3.00	0.000
Anterior TSD (%)	78.38 (3.00)	70.21	83.92	77.24 (2.5)	69.52	81.48	0.038*
Overall TSD (%)	91.32 (2.5)	79.23	99.47	90.92 (1.90)	78.86	100.21	0.37
Posterior TSD (%)	12.94 (1.50)	9.42	15.63	13.68 (1.40)	10.29	16.14	0.015*

*Note*: Normal TSD ratios: anterior TSD: 77.2 (1.65) %; overall TSD: 91.3 (1.91) %; posterior TSD: 14.1%. *p* value according to independent *t* test.

Abbreviations: AOB, anterior open bite; TSD, tooth size discrepancy.

* *p* < .05.

As shown in Table 3, in the total sample, a significant difference between males and females was found in the posterior TSD ratio with a mean of 12.89% in males and 13.61% in females (*p* = .02). On the other hand, no significant difference between genders was found in anterior or overall TSD ratios (*p* ˃ .05). In the control group, a significant difference was also found between males and females in the posterior TSD ratio with a mean of 13.04% in males and 14.03% in females (*p* = .02). No significant differences were detected in the other TSD ratios (*p* > .05). On the other hand, in the AOB group, no significant differences were detected between males and females in any of the TSD ratios (*p* > .05).

**Table 3:** Means and standard deviations of TSD ratios according to gender in the total sample, skeletal AOB, and control groups

	Male Mean (SD)	Female Mean (SD)	*p* Value
Total sample			
Anterior TSD	78.34% (3.03)	77.43% (2.70)	0.10
Overall TSD	91.24% (2.10)	91.03% (2.34)	0.66
Posterior TSD	12.89% (1.50)	13.61% (1.71)	0.02*
AOB group			
Anterior TSD	79.03 (3.00)	77.80 (3.04)	0.15
Overall TSD	91.80 (1.91)	90.88 (2.90)	0.19
Posterior TSD	12.80 (1.71)	13.10 (1.40)	0.48
Control group			
Anterior TSD	77.44 (2.34)	77.13 (3.40)	0.67
Overall TSD	90.50 (2.13)	91.20 (1.81)	0.24
Posterior TSD	13.04 (1.20)	14.03 (1.50)	0.02*

*Note*: Normal TSD ratios: anterior TSD: 77.2 (1.65)%; overall TSD: 91.3 (1.91)%; posterior TSD: 14.1%. *p* value according to independent *t* test.

Abbreviations: AOB, anterior open bite; TSD, tooth size discrepancy.

* *p* < .05.

The mean (SD) AOB measurement in the AOB group was −4.50 (−1.40) mm. No correlation was found between the amount of AOB and the three TSD ratios; (*p* > .05) as shown in Table 4.

**Table 4:** Correlation between amount of AOB and TSD (anterior, posterior, and overall) ratios

AOB value	Anterior TSD	Posterior TSD	Overall TSD
Pearson's correlation coefficients	−0.21	0.09	−0.20
*p* value	0.15	0.54	0.20

Abbreviations: AOB, anterior open bite; TSD, tooth size discrepancy.

## 4 DISCUSSION

This study was retrospective in nature and aimed to find out if there is any relationship between TSD and skeletal AOB in Class I malocclusion patients. Also, the correlation between the amount of AOB and the TSD was assessed.

The measurements were carried out directly on the study models using a digital caliper. A digital caliper was used extensively in previous studies and proved to be accurate and valid (Al‐Khateeb & Abu Alhaija, 2006; Al‐Omari et al., 2008).

Berger and Janisse distinguished between dental and skeletal open bite in terms of diagnostic features and etiological factors. They concluded that when a divergent MMPA exists, the open bite can be classified as skeletal (Berger & Janisse, 2013). Also, in the current study, only those cases with a PFH to AFH ratio of less than 59% were included to ensure the skeletal nature of AOB.

In the present study, 50 cases of pretreated skeletal AOB patients were chosen when more than 3 mm of AOB and skeletal nature (MMPA > 32°, PFH/AFH< 59%) were detected. All cases in both groups were chosen to be an Angle Class I relationship, in order to eliminate any effect from different skeletal and dental relationships since TSD was found to be significantly different in different malocclusion types (Alkofide & Hashim, 2002; Nie & Lin, 1999; Sperry et al., 1977; Uysal et al., 2005).

In the present study, the results demonstrated a significant difference between the AOB group and the normal overbite group in the anterior TSD ratio as well as the posterior ratio. However, the differences were less than 1 SD of Bolton's ratios. This means that although statistically, the differences were significant, clinically they were not.

Anterior TSD ratio was higher in the AOB patients than in those with a normal overbite, which means either the mandibular anterior teeth are larger mesio‐distally (M‐D) or the upper anterior teeth are smaller M‐D in the AOB patients; a recommendation of reducing the mandibular anterior teeth width (e.g., interdental stripping) or adding on the maxillary anterior teeth (restorative approach) could be considered for AOB patients depending on the diagnosed case clinically.

On the other hand, the posterior TSD ratio was lower in the AOB patients, which means either the maxillary posterior teeth are larger M‐D or the mandibular posterior teeth are smaller M‐D; the opposite treatment approach could be done in this case for AOB patients (restorative addition in lower teeth or reduction of upper teeth width).

On the other hand, the overall TSD was not found significantly different between skeletal AOB and normal overbite groups. This can be expected as a result of the cancellation effect as the anterior TSD ratio was found significantly higher whereas the posterior TSD ratio was found lower in the AOB group.

Our results are different from that reported by Taibah (Salwa, 2016), who found no differences between AOB and control groups in any of the TSD ratios. This difference may be related to the nature of the AOB studied. In our study only skeletal AOB cases were included whereas in Taibah's study, they looked at the study models indicating that they included dental AOB only. Also, Taibah (Salwa, 2016) did not mention in her study the type of malocclusion included. In the current study, however, only Class I dental and skeletal malocclusion was included to eliminate the effect of different skeletal and dental relationships since TSD was found to be significantly different in different malocclusion types and skeletal relationships (Alkofide & Hashim, 2002; Nie & Lin, 1999; Sperry et al., 1977; Uysal et al., 2005).

Many authors through the literature went through the correlation between the anterior−posterior (A−P) relationship and TSD. Angle's Class III was found to have the highest overall TSD ratio (Sperry et al., 1977) and the anterior TSD ratio (Alkofide & Hashim, 2002), which means a relative tooth size excess in the mandibular teeth, whereas the lowest ratios were found in Angle's Class II cases (Nie & Lin, 1999), which reflects a relative maxillary tooth excess.

Grauer and coworkers have mentioned some of the clinical findings that could be related to TSD (Grauer et al., 2012). The excessive or deficient overbite was one of the occlusal abnormalities that could be detected with TSD. Excessive prominence of the upper incisors and canine marginal ridges, and abnormally angulated or inclined incisors and canines were also found in patients with TSD.

A genetic influence could be another explanation for our findings where TSD and skeletal AOB were both related to genetic and environmental factors in many studies throughout the literature. Genetic influences have been considered important in the determination of tooth dimensions, and the first report on this subject was published by Horowitz and his colleagues in a twin study (Horowitz et al., 1958). They reported that a greater tooth size correlation was found in monozygotic twins. Stewart and Prescott suggested that the tooth size factor is multifactorial, with the environment playing an important role in addition to the genetic contribution (Stewart & Prescott, 1979).

In the current study, a significant difference between males and females was found in the posterior TSD ratio only. This was detected in the total sample and normal bite group with no significant differences in anterior or overall TSD ratios. In the AOB group, on the other hand, no significant gender differences were detected in any of the TSD ratios.

Most studies looked at anterior and overall TSD ratios and missed the posterior TSD ratio since it was not mentioned originally by Bolton (Bolton, 1958, 1962). In this study, in addition to anterior and overall TSD ratios, the posterior TSD ratio was also assessed.

It has been suggested that males have larger teeth compared to females (Bishara et al., 1989); however, differences in TSD ratios are different from differences in absolute tooth size.

Various studies have detected gender differences in the intermaxillary TSD ratios (Al‐Khateeb & Abu Alhaija, 2006; Malkoç et al., 2011), whereas other studies found no such differences (Al‐Omari et al., 2008; Sharma et al., 2011).

Lavelle compared TSD ratios between males and females and found that males have larger overall and anterior TSD ratios compared to females. However, these differences were small (Lavelle, 1972). Another study supported that males have larger anterior and overall TSD ratios although the differences were less than 1 SD from Bolton's ratios. Richardson and Malhotra, on the other hand, found no significant gender differences in anterior and posterior TSD ratios (Richardson & Malhotra, 1975).

With regard to the correlation between the amount of AOB and TSD, it has not been studied before, so a comparison with other studies could not be done. In this study, the correlation between the amount of AOB and TSD ratios was not significant. Although we found that anterior and posterior TSD ratios were significantly different in the skeletal AOB group compared to the normal bite group, the amount of the AOB could not be used as a predictor for the TSD.

## 5 LIMITATIONS OF THE STUDY

- Relatively small sample size, the overall number of the studied sample was 100 cases, taking into consideration the difficulty in collecting skeletal AOB cases with full records including good quality study models.

## 6 CONCLUSIONS

1. Anterior TSD ratio was higher in skeletal AOB patients compared to normal bite patients.
2. Posterior TSD ratio was less in skeletal AOB patients compared to normal bite cases.
3. The overall TSD ratio showed no significant difference between the two groups.
4. Significant difference between males and females was found in the posterior TSD ratio only, males showed less ratio compared to females.
5. No direct correlation was found between the amount of AOB and the three TSD ratios: anterior, posterior, and overall.

## AUTHOR CONTRIBUTIONS

All authors contributed to the study. **Emad F. Al Maaitah**: Conception and design; data analysis; writing—original draft preparation; writing—review and editing; funding acquisition; supervision. **Nada Al‐Madani**: Data collection; materials and methods; writing—first draft. **Elham S. Abu Alhaija**: Data analysis; writing—review and editing; supervision.

## CONFLICT OF INTEREST

The authors declare no conflict of interest.

## Data Availability

The data that support the findings of this study are available on request from the corresponding author. The data are not publicly available due to privacy or ethical restrictions.
